# Effects of Kampo on functional gastrointestinal disorders

**DOI:** 10.1186/1751-0759-8-5

**Published:** 2014-01-21

**Authors:** Takakazu Oka, Hirokuni Okumi, Shinji Nishida, Takashi Ito, Shinichi Morikiyo, Yoko Kimura, Masato Murakami

**Affiliations:** 1 Department of Psychosomatic Medicine, Graduate School of Medical Sciences, Kyushu University, Fukuoka 812-8582, Japan; 2 Department of Medical Oncology, Division of Psychosomatic Medicine, Kinki University, Faculty of Medicine, Osaka 589-8511, Japan; 3 Department of Psychosomatic Medicine, Japanese Red Cross Wakayama Medical Center, Komatsubara-dori 4-20, Wakayama 640-8558, Japan; 4 Center of Japanese Oriental Medicine, Kashima Rosai Hospital, Labour Welfare Corporation, Doaihoncho 1-9108-2, Kamisu-shi, Ibaraki 314-0343, Japan; 5 Department of Psychosomatic Medicine and Psychiatry, Gujo City Hospital, 1261 Shimadani, Hachiman-cho, Gujo City, Gifu 501-4222, Japan; 6 Institute of Oriental Medicine, Tokyo Women’s Medical University, 1-21-8 Tabata, Kita-ku, Tokyo 114-0014, Japan; 7 Department of Psychosomatic Medicine, Nihon University Itabashi Hospital, Oyaguchikamicho 30-1, Itabashi-ku, Tokyo 173-0032, Japan

**Keywords:** Kampo, Rikkunshito, Keishikashakuyakuto, Functional dyspepsia, Irritable bowel syndrome

## Abstract

This article reviews the effectiveness of Kampo (traditional Japanese herbal medicine) in the treatment of functional gastrointestinal disorders, especially functional dyspepsia (FD) and irritable bowel syndrome (IBS). The results of four randomized, controlled trials (RCTs) suggested the usefulness of rikkunshito in relieving the subjective symptoms of patients with FD. Rikkunshito significantly improved not only gastric symptoms, such as epigastiric discomfort, but also extra-gastric symptoms, such as general fatigue, when compared with control drugs. The therapeutic effects of rikkunshito were more evident when it was prescribed to patients with *“kyosho”*, i.e., low energy. Two RCTs suggested the efficacy of keishikashakuyakuto for IBS.

Basic research studies have demonstrated that these Kampo medicines have multiple sites of action to improve subjective symptoms. For example, rikkunshito improves gastric motility dysfunction, including impaired adaptive relaxation and delayed gastric emptying, gastric hypersensitivity, and anorexia via facilitation of ghrelin secretion. It also exhibits anti-stress effects, i.e., it attenuates stress-induced exacerbation of gastric sensation and anorexia, as well as the hypothalamic-pituitary-adrenocortical axis and sympathetic activation. Keishikashakuyakuto exhibited not only an antispasmodic effect on intestinal smooth muscle, but also antidepressant-like effects. Case series suggest that other Kampo prescriptions are also effective for FD and IBS. However, further studies are necessary to evaluate their efficacy.

## 

Kampo refers to Japanese traditional medicine, including herbal medicine. In Japan, herbal medicine is very popular, and it is covered by national health insurance. Psychosomatic medicine specialists often prescribe Kampo medicines to treat functional somatic diseases, such as functional gastrointestinal disorders (FGIDs). Patients with FGIDs complain of symptoms originating from the gastrointestinal tract without evident organic diseases. These symptoms are bothersome for patients and difficult to treat, thus decreasing patient quality of life. The pathophysiology of FGIDs is multifactorial. Both physiological factors, including abnormal motility, visceral hypersensitivity, inflammation, and altered bacterial flora, as well as psychosocial factors, including psychological stress, psychological state, coping style, and social support affect brain-gut interactions and clinical outcomes [1,2].

Currently, many Kampo formulas are prescribed to treat FGIDs. However, the evidence of their efficacy is limited. Furthermore, as many earlier studies conducted in the 1980’s and 90’s were published in Japanese, those pioneering works have not been introduced into the English literature or indexed in PubMed.

Therefore, the aims of this paper are (1) to review the clinical trials that investigated the effect of Kampo on FGIDs, including studies published in Japanese, and (2) to provide an overview of the basic mechanisms as to how Kampo improves the subjective symptoms of patients with FGIDs, focusing on functional dyspepsia (FD) and irritable bowel syndrome (IBS).

## Methods

We performed literature searches on PubMed and Ichushi Web (papers written in Japanese) on June 30, 2013 using the keywords Kampo (or each prescription name) and functional dyspepsia or irritable bowel syndrome. We included reports regarding Kampo extract products that comply with the formulation standards established in 1986 and excluded those regarding drug solutions of crude drug pieces for decoction, powdered crude drugs, and over the counter (OTC) products. We abstracted clinical studies conducted as randomized, controlled trials (RCTs) and case series that involved at least 10 cases. We present the results descriptively, because a meta-analysis was not possible due to the limited number of RCTs. We also introduce basic research findings to explain the possible mechanisms of action of each Kampo medicine.

## Results

### Functional dyspepsia

To date, four RCTs, including one double blind (DB)-RCT and 13 case series have been published. All studies were conducted in Japan. Most of them, especially all of the RCTs, studied the effectiveness of rikkunshito on dyspeptic symptoms. In these studies, the diagnosis of FD was based on either the American Gastroenterology Association working party criteria, in which the condition is named non-ulcer dyspepsia [3], or the Rome III diagnostic criteria, in which the condition is named FD [4]. In this analysis, we included both.

#### Efficacy of rikkunshito

According to the traditional medicine point of view, rikkunshito is a Kampo medicine for treating upper gastrointestinal symptoms, such as nausea, indigestion, and anorexia in patients with “*kyo-sho*”, i.e., subjects who have weak constitutions and low vital energy. Rikkunshito extract granules for ethical use (Tsumura and Co., Product number TJ-43) (7.5 g) contain 4.0 g of dried extract obtained from mixed raw herbs in the following ratio: Atractylodes Lancea Rhizome, 4.0 g; Ginseng, 4.0 g; Pinellia Tuber, 4.0 g; Poria Sclerotium, 4.0 g; Jujube, 2.0 g; Citrus Unshiu Peel, 2.0 g; Glycyrrhiza, 1.0 g; and Ginger, 0.5 g.

Miyoshi and colleagues conducted the first multi-center RCT to compare the therapeutic effects of rikkunshito and cisapride, a prokinetic drug, on the digestive symptoms of patients with non-ulcer dyspepsia. Subjects were randomized to receive either oral treatment with 2.5 g rikkunshito three times daily (rikkunshito group, 111 subjects) or cisapride (2.5 mg) three times daily (cisapride group, 104 subjects) for four weeks. Significantly greater improvement of upper gastrointestinal symptoms, especially epigastralgia and abdominal discomfort, was found for rikkunshito than for cisapride [5]. Rikkunshito also resulted in greater, but non-significant, improvement of extra-gastric symptoms, such as coldness in the lower extremities, dizziness, and appetite loss when compared with cisapride. The global improvement rate of the rikkunshito group was higher than that of the cisapride group when this Kampo medicine was prescribed to subjects whose constitutions were weak or who felt moderate general fatigue, i.e., subjects with “*kyo-sho*”.

Harasawa and coworkers conducted a multi-center DB-RCT to assess the effect of rikkunshito on dismotility-like dyspepsia [6], one subtype of FD defined by the Rome II diagnostic criteria. Subjects were randomized to receive either oral treatment with 2.5 g rikkunshito three times daily (rikkunshito group, 133 subjects) or 2.5-g granules containing a low dose (2.5%) of rikkunshito three times daily as a control (control group, 117 subjects) for two weeks. Rikkunshito significantly improved the dysmotility-like dyspepsia generalized improvement rate (DDGIR) and subjective symptoms, including epigastric discomfort, anorexia and, interestingly, general fatigue [7] when compared with the control group. In the rikkunshito group, the DDGIR improved more in patients whose vigor or physical strength was low when compared with those whose energy or strength was normal. The incidence of adverse events was not different between the two groups, i.e., adverse events were reported by seven subjects in the rikkunshito group and seven subjects in the control group. The most frequently observed adverse symptom was diarrhea (3 subjects in each group). The results of these two RCTs suggested the effectiveness of rikkunshito for FD. Furthermore, these studies also suggested that rikkunshito is more effective when it is prescribed to subjects with *“kyo-sho”*, based on the traditional medicine diagnosis in patients with FD. These findings were published in Japanese.

Tatsuta and colleagues investigated the effects of rikkunshito on gastric emptying as well as the gastrointestinal symptoms of 42 dyspeptic patients in a RCT and first reported their findings in English [8]. In this study, by random allocation, 22 patients received oral treatment with 2.5 g rikkunshito three times daily, and 20 patients received combizym as a placebo. Gastric emptying was measured by the acetaminophen absorption method. After seven days of treatment, gastric emptying was significantly accelerated, and gastrointestinal symptoms, such as epigastric fullness, heartburn, belching, and nausea, were significantly improved in patients treated with rikkunshito. In contrast, placebo treatment produced no significant effects on gastric emptying and subjective symptoms.

Arai and colleagues compared the effects of four weeks of treatment with rikkunshito or domperidone, a prokinetic drug, on the gastrointestinal symptoms of 27 patients with FD in a RCT and found that dyspeptic symptoms improved significantly in both groups [9].

The results of these four RCTs indicate that rikkunshito is a useful drug for treating FD. Two RCTs suggested that the therapeutic effect of rikkunshito on FD was stronger when it is prescribed to subjects with “*kyo-sho*”, based on the traditional medicine diagnosis. There were 13 case series. Most of them reported the effectiveness of rikkunshito. Other studies suggested the effectiveness of hangekobokuto [10,11] and hangeshashinto. Notably, two studies suggested that hangeshashinto is effective for dyspeptic patients for whom in rikkunshito was not effective [12,13].

#### Therapeutic mechanisms of rikkunshito

The subjective symptoms of FD are attributed to disturbed gastric motility and gastric hypersensitivity [4,14]. Psychological stress affects gastric motility [15] and gastric sensitivity [16,17] and causes or exacerbates FD symptoms [18]. Some patients with FD suffer from co-morbid psychiatric disorders, such as depressive and anxiety disorders [18-20]. Any or all of these components may contribute to the development of subjective symptoms in patients with FD [20,21]. Human and animal studies have shown that rikkunshito has effects on each of these factors.

#### Gastric motility dysfunction

In patients with FD, gastric motility functions, such as gastric adaptive relaxation (or gastric accommodation) and gastric emptying, are impaired.

#### Gastric emptying

Harasawa and coworkers have demonstrated that a two-week administration of rikkunshito accelerated gastric emptying, as assessed by the acetaminophen absorption method, and improved upper gastrointestinal symptoms in patients with FD whose gastric emptying was delayed [22]. Tatsuta and coworkers also reported that rikkunshito improved gastric emptying in patients with FD [8]. Animal studies have suggested that rikkunshito accelerates gastric emptying [8,22] via nitric oxide-mediated mechanisms [23] and antagonism of 5-HT3 receptors [24].

#### Adaptive relaxation

Two weeks of rikkunshito administration significantly expanded the proximal stomach after incremental ingestion of a liquid meal up to 400 mL in patients with FD, suggesting that rikkunshito promotes gastric adaptive relaxation [25], a finding that had been observed in animal models [26,27].

#### Other motility functions

Rikkunshito was also reported to coordinate gastric myoelectric activity in patients with dyspeptic symptoms after gastrointestinal surgery [28] and to stimulate gastrointestinal motility in dogs [29,30].

#### Gastric hypersensitivity

The effect of rikkunshito on gastric hypersensitivity has not yet been investigated in patients with FD. However, in healthy subjects, rikkunshito was reported to reduce epigastric fullness following gastric distension via a gastric barostat. Rikkunshito also attenuated psychological stress-induced reduction of gastric volume at the sensory threshold and stress-induced increased anxiety at the discomfort threshold [31], suggesting that rikkunshito improves gastric symptoms under distension, possibly by affecting gastric sensorimotor function.

#### Ghrelin secretion

Ghrelin is a 28-amino acid orexigenic peptide found in the stomach [32]. Takeda and colleagues demonstrated that rikkunshito suppresses cisplatin-induced decreases in plasma levels of acylated ghrelin, an active form of ghrelin, and that it increases food intake via serotonin (5-HT) 2B/2C receptor antagonism in rats [33]. After that report, research intensified to determine whether rikkunshito increases ghrelin secretion in human subjects, leading to improvement of appetite and dyspeptic symptoms (for review, see [34]). Oral administration of rikkunshito for two or four weeks has been demonstrated to increase plasma levels of acylated ghrelin in healthy subjects [35], patients with gastric cancer [36], and patients with FD [9]. Arai and coworkers compared the effects of rikkunshito with the effect of domperidone on plasma ghrelin levels and gastrointestinal symptoms in patients with FD. Four weeks after treatment, the plasma level of acylated ghrelin in the rikkunshito group was significantly higher than that in the domperidone group, whereas the plasma level of desacylated ghrelin, an inactive form of ghrelin, was not different between the two groups [9]. Furthermore, in the rikkunshito group, the improvements in reflux and indigestion showed positive correlations with the increase in acylated ghrelin. Among the eight ingredients of rikkunshito, flavonoids of citrus unshiu peel, i.e., heptamethoxyflavone and hesperidin, and flavonoids of glycyrrhiza, i.e., isoliquiritigenin have 5-HT_2B_ antagonist activity and reverse the cisplatin-induced suppression of plasma acylated ghrelin [33]; and atractylodin, a component of the Atractylodes Lancea Rhizome, potentiates ghrelin receptor signaling [37].

These findings suggest that rikkunshito improves the subjective symptoms of FD, at least in part, by facilitating the secretion of ghrelin.

#### Psychological factors and stress

We assessed the effect of 4 weeks of rikkunshito administration on depressive symptoms in patients with FD. Rikkunshito significantly decreased depression scores, as assessed by the Self-Rating Questionnaire for Depression [38]. As depressed patients frequently show high serum cortisol levels, we also investigated the effect of four weeks of rikkunshito administration on serum cortisol levels at 9 a.m. in patients with FD [39]. Seven of 23 patients showed levels higher than the normal range. Four weeks of rikkunshito administration significantly decreased their cortisol levels, whereas rikkunshito did not affect the cortisol levels of patients with levels in the normal range. An animal study has demonstrated that rikkunshito attenuates the activities of corticotropin-releasing hormone (CRH)-producing neurons and reduces anxiety-like behavior in tumor-bearing rats. As cancer-induced anorexia and cachexia are induced by 5-HT_2c_ receptor-mediated interactions of 5-HT with CRH-producing neurons and CRH activates the hypothalamic-pituitary-adrenocortical (HPA) axis, decreases the plasma levels of acylated ghrelin, and induces anxiety-like behavior, the inhibitory effect of rikkunshito on CRH-producing neurons may contribute to psychological effects and normalization of high cortisol levels [37].

Rikkunshito was also shown to attenuate stress-induced physiological responses. For example, rikkunshito attenuated acute stress-induced increases in plasma levels of adrenocorticotropic hormone, cortisol [40], and neuropeptide Y [41] evoked by repeated blood sampling in healthy subjects. As neuropeptide Y and noradrenaline are coreleased from the sympathetic nerve terminals by activation of the sympathetic nervous system and are released into plasma during intensive sympathetic activation, neuropeptide Y is considered a marker of stress-induced sympathetic activation.

In addition to its inhibitory effect on the stress-induced activation of the HPA axis and the sympathetic nervous system, rikkunshito improved the delayed gastrointestinal tract motility induced by stress mimicking a Japanese summer, i.e., high temperature and humidity stress in mice [42]. Rikkunshito also reversed the stress-induced reduction of intragastric volume stimulated by a barostat at the sensory threshold in healthy subjects [31]. Furthermore, rikkunshito reversed the novelty stress-induced reduction in food intake via ghrelin secretion in rats [43]. Based on these findings, “A Guideline for the Diagnosis and Treatment of Psychosomatic Diseases 2006” recommends prescription of rikkunshito for treating FD [44]. Furthermore, rikkunshito is widely prescribed for treating the upper gastrointestinal tract symptoms of patients with gastroesophageal reflux disease [45-47], gastric cancer [36], patients who underwent gastrointestinal surgery [28] or chemotherapy [48,49], and patients who take selective serotonin reuptake inhibitors [50]. Rikkunshito is also thought to be a promising drug for ameliorating anorexia-cachexia in patients with cancer [37].

### Irritable bowel syndrome

To date, two RCTs, including one DB-RCT and 14 case series have been published. All studies were conducted in Japan and published in Japanese. Two RCTs studied the effectiveness of keishikashakuyakuto on IBS [51,52].

#### Efficacy of keishikashakuyakuto

Keishikashakuyakuto is a Kampo medicine that is prescribed to relieve abdominal pain. Keishikashakuyakuto extract granules for ethical use (Tsumura and Co., Product number TJ-60) (7.5 g) contain 3.75 g of dried extract obtained from mixed raw herbs in the following ratio: Peony root, 6.0 g; Cinnamon Bark, 4.0 g; Jujube Fruit, 4.0 g; Glycyrrhiza Root, 2.0 g; and Ginger Rhizome, 1.0 g.

Mizuno and colleagues conducted the first RCT to compare the therapeutic effect of keishikashakuyakuto with mepenzolate bromide, an anti-cholinergic agent, on IBS. Subjects were randomized to receive either oral treatment with 2.5 g keishikashakuyakuto three times daily (keishikashakuyakuto group, 26 subjects) or mepenzolate bromide (15 mg) three times daily (mepenzolate group, 24 subjects) for eight weeks. At four weeks after the start of treatment, the keishikashakuyakuto group had significantly greater improvement than mepenzolate of IBS symptoms, including stool abnormality, abdominal pain, gas symptoms, and rumbling stomach. The final overall improvement rate was significantly higher in the keishikashakuyakuto group than in the mepenzolate group [51].

Sasaki and coworkers conducted a multi-center DB-RCT to investigate the effect of keishikashakuyakuto. Subjects were randomized to receive either oral treatment with 2.0 g keishikashakuyakuto (EK-60, Kracie and Co.) three times daily (keishikashakuyakuto group, 124 subjects) or 2.0-g granules containing a low dose (5%) of keishikashakuyakuto three times daily (low-dose group, 108 subjects) for eight weeks. The final overall improvement rate was not different between the two groups. However, the percentage of patients with improved abdominal pain was significantly higher in the keishikashakuyakuto group (58% improved moderately or more) than in the low-dose group (37%) in patients with the diarrhea-predominant type IBS. Such a difference was not found in patients with the constipation-predominant type or alternating type IBS. This study suggests that keishikashakuyakuto is effective for relieving abdominal pain in patients with the diarrhea-predominant type IBS [52].

Among 14 case studies of IBS, most studied the effectiveness of keishikashakuyakuto. Others investigated the effectiveness of other Kampo formulas and suggested their usefulness. Such prescriptions included saireito, keihito, heiisan, and daikenchuto. Oka and colleagues found that, in spite of favorable improvement of abdominal symptoms such as abdominal pain or diarrhea (percentage of moderate improvement or more two weeks after treatment, >80%), keishikashakuyakuto was less effective for extra-colonic symptoms, such as general fatigue or appetite loss (50-60%). They demonstrated that Kampo therapy became more effective for relieving the extra-colonic symptoms of IBS patients who did not respond to keishikashakuyakuto when prescribed based on the traditional medicine diagnosis, considering the dimensions of “ki-ketsu-sui” or “coldness-heat” balances [53].

#### Therapeutic mechanisms of keishikashakuyakuto

Peony root and Glycyrrhiza root, both of which are the main crude ingredients of keishikashakuyakuto, suppress the neurogenic contractions of the ileum induced by electrical stimulation and ganglionic-stimulating agents, such as dimethylphenylpiperazinium and nicotine in guinea pigs via inhibition of acetylcholine (Ach) release from cholinergic nerves and inhibition of Ach action on ileum smooth muscle [54]. Their antispasmodic effect was also confirmed for the human colon [55]. In addition to its spasmolytic and smooth muscle-relaxing effects [56-58], peony root has anti-inflammatory and analgesic effects and inhibits gastric acid secretion and stress-induced ulceration [59]. Furthermore, peony root was demonstrated to have sedative [60] and antidepressant-like effects in mice [61-66]. The antidepressant-like effect of peony root could be, at least in part, mediated by inhibiting monoamine oxidase activity, hypothalamic-pituitary-adrenal axis activation, oxidative stress, and up-regulated brain-derived neurotrophic factor [62-66].

## Discussion

We reviewed RCTs that evaluated the effects of Kampo medicines on FGIDs. Clinical trial results have suggested that several Kampo medicines are useful for relieving the subjective symptoms of patients with FGIDs, i.e., rikkunshito for FD and keishikashakuyakuto for IBS.

In addition to efficacy, these studies suggest several interesting characteristics of Kampo as pharmacotherapy. First, rikkunshito was effective not only for gastrointestinal symptoms but also for the extra-gastric symptoms of patients with FD, such as dizziness, coldness in the lower extremities, or general fatigue. As patients with FGIDs are known to complain of many symptoms that are not of gastrointestinal origin [67,68], this characteristic of Kampo medicine may be beneficial for improving the quality of life of these patients. Second, basic research has demonstrated that Kampo medicines have multiple sites of action to alleviate symptoms. As was described previously, Kampo medicines act on both the central nervous system (mind) and the gastrointestinal tract (body) to relieve symptoms. They also exhibit anti-stress effects. These multiple actions could be beneficial for patients with FGID and comorbid psychiatric disorders, patients whose physical symptoms are modulated by psychological factors such as anxiety or depression, or patients whose symptoms are exacerbated by psychological stress (Table 1). Third, Kampo medicines, at least rikkunshito and keishikshakuyakuto, become more effective if patients are diagnosed and treated based on the viewpoint of traditional medicine. This is reasonable because Kampo medicines were originally prescribed based on a traditional medicine diagnosis, e.g. *“kyo-sho”*, not on the modern medicine diagnosis, e.g. FD. To accumulate evidence for the efficacy of Kampo medicine, it is necessary to conduct RCTs to evaluate its efficacy based on modern medicine diagnoses. In the future, however, clinical studies should be conducted based on both approaches, like the studies by Miyoshi [5] and Harasawa [6].

**Table 1:** Beneficial effects of Kampo medicines on mind, stress, and body in functional gastrointestinal disorders

	**Effects on brain**	**Effects on stress**	**Effects on GI tract**
Rikkunshito	Reduces depressive symptoms and high cortisol levels.	Inhibits stress-induced activation of HPA axis and SNS.	Improves gastric motility dysfunction, i.e., gastric emptying and gastric accommodation.
	Reduces anxiety.		
	Inhibits activities of CRH-producing neurons.		Modulates gastric hypersensitivity.
			Increases ghrelin secretion.
Keishikashakuyakuto	Has sedative, antidepressant-like effects.	Inhibits stress-induced activation of HPA axis.	Has anti-spasmodic, anti-inflammatory, and analgesic effects.
	Increases BDNF		

BDNF, brain-derived neurotrophic factor; CRH, corticotropin-releasing hormone; GI, gastrointestinal; HPA, hypothalamic-pituitary-adrenocortical; SNS, sympathetic nervous system.

Limitations: Case series have suggested that hangekobokuto and hangeshashinto are effective for FD and that saireito, keihito, heiisan, and daikenchuto are effective for IBS. Their mechanisms of action are also well studied and account for their effects. For example, hangekobokuto was demonstrated to facilitate gastric emptying [10] and decrease the bowel gas [11] of patients with FD. However, so far, the effects of these Kampo medicines have not been evaluated in RCTs. Further studies are necessary to determine whether these Kampo medicines are really effective for FGIDs.

Recently, the gastroenterologists Tominaga and Arakawa published an excellent review article on Kampo for gastrointestinal disorders [69]. The strengths of their paper are that they reviewed the effects of Kampo on a broader range of disorders than we introduced in this article, including gastroesophageal reflux disorders and post-operative ileus, and that they described the basic mechanisms of action of Kampo on the gastrointestinal tract more precisely than we did. The strengths of our review article are that we introduced the RCTs and case series written in Japanese, to which Tominaga and Arakawa did not refer in their article, because we thought it important to introduce such pioneering works and that we described the effects of Kampo on extra-gastrointestinal tract symptoms and on the central nervous system and stress systems as well as on the gastrointestinal tract from the psychosomatic medicine point of view. Therefore, these two review articles will enable readers to understand the whole picture of the role of Kampo as a treatment for FGIDs.

## Conclusions

This article reviewed the effects of Kampo medicines on FGIDs, focusing on FD and IBS. Four RCTs suggested the usefulness of rikkunshito for FD. Two RCTs suggested the usefulness of keishikashakuyakuto for IBS. These Kampo medicines act on both the brain and the gastrointestinal tract to alleviate subjective symptoms. Case series suggest that several other Kampo medicines are also effective for FGIDs. However, further studies are necessary to evaluate their efficacy.

## Competing interests

The authors declare that they have no competing interests.

## Authors’ contributions

TO designed the study protocol, analyzed the data, and drafted the manuscript. HO reviewed “functional dyspepsia” and SN reviewed “irritable bowel syndrome”. TI, SM, YK, and MM discussed the data and manuscript. All authors contributed to this project as members of Japanese Association of Oriental Psychosomatic Medicine, Evidence-Based Medicine Working Team (JOPM-EBM). All authors read and approved the final manuscript.
